# Characterization of genome-reduced *Bacillus subtilis* strains and their application for the production of guanosine and thymidine

**DOI:** 10.1186/s12934-016-0494-7

**Published:** 2016-06-03

**Authors:** Yang Li, Xujun Zhu, Xueyu Zhang, Jing Fu, Zhiwen Wang, Tao Chen, Xueming Zhao

**Affiliations:** Key Laboratory of Systems Bioengineering (Ministry of Education), SynBio Research Platform, Collaborative Innovation Center of Chemical Science and Engineering (Tianjin), School of Chemical Engineering and Technology, Tianjin University, Tianjin, 300072 China; Hubei Provincial Cooperative Innovation Center of Industrial Fermentation, Key Laboratory of Fermentation Engineering (Ministry of Education), Hubei University of Technology, Wuhan, 430068 China; Tianjin Vocational College of Bioengineering, Tianjin, 300462 China; College of Life Science, Shihezi University, Shihezi, 832000 China

**Keywords:** *Bacillus subtilis*, Genome reduction, Chassis cell, Guanosine, Thymidine, Nucleosides

## Abstract

**Background:**

Genome streamlining has emerged as an effective strategy to boost the production efficiency of bio-based products. Many efforts have been made to construct desirable chassis cells by reducing the genome size of microbes. It has been reported that the genome-reduced *Bacillus subtilis* strain MBG874 showed clear advantages for the production of several heterologous enzymes including alkaline cellulase and protease. In addition to enzymes, *B. subtilis* is also used for the production of chemicals. To our best knowledge, it is still unknown whether genome reduction could be used to optimize the production of chemicals such as nucleoside products.

**Results:**

In this study, we constructed a series of genome-reduced strains by deleting non-essential regions in the chromosome of *B. subtilis* 168. These strains with genome reductions ranging in size from 581.9 to 814.4 kb displayed markedly decreased growth rates, sporulation ratios, transformation efficiencies and maintenance coefficients, as well as increased cell yields. We re-engineered the genome-reduced strains to produce guanosine and thymidine, respectively. The strain BSK814G2, in which *purA* was knocked out, and *prs*, *purF* and *guaB* were co-overexpressed, produced 115.2 mg/L of guanosine, which was 4.4-fold higher compared to the control strain constructed by introducing the same gene modifications into the parental strain. We also constructed a thymidine producer by deleting the *tdk* gene and overexpressing the *prs*, *ushA*, *thyA*, *dut*, and *ndk* genes from *Escherichia coli* in strain BSK756, and the resulting strain BSK756T3 accumulated 151.2 mg/L thymidine, showing a 5.2-fold increase compared to the corresponding control strain.

**Conclusions:**

Genome-scale genetic manipulation has a variety of effects on the physiological characteristics and cell metabolism of *B. subtilis*. By introducing specific gene modifications related to guanosine and thymidine accumulation, respectively, we demonstrated that genome-reduced strains had greatly improved properties compared to the wild-type strain as chassis cells for the production of these two products. These strains also have great potential for the production of other nucleosides and similar derived chemicals.

**Electronic supplementary material:**

The online version of this article (doi:10.1186/s12934-016-0494-7) contains supplementary material, which is available to authorized users.

## Background

In recent years, there has been considerable interest in biotechnology and synthetic biology research concerning the design and construction of an optimal chassis cell [1]. Genome streamlining is an effective strategy for obtaining promising and robust host cells [2]. Genome reduction is a universal phenomenon in symbiotic bacteria and certain other bacteria that inhabit highly specialized habitats [3]. Inspired by these natural evolutionary events, researchers proposed that an ideal chassis cell can be generated by genome engineering and accelerated evolution [4]. Bacterial genomes show great diversity in size and composition, and this diversity is the result of millions of years of horizontal gene transfer and other complex evolutionary events [5, 6]. It is expected that the removal of a number of non-essential regions from a bacterial chromosome will facilitate the optimization of metabolic pathways and energy utilization by enhancing the predictability of genetic engineering, which can improve overall metabolic performance [7]. Based on this vision, studies have been conducted to streamline genomes and build minimized cell factories for the production of a variety of drugs, biofuels and other bio-based chemicals [8–10].

Previous studies revealed that genome reduction resulted in beneficial traits such as genotypic stability and phenotypic validity. For example, the *Escherichia coli* strain MDS42, that lacks 14.3 % of the parent strain’s genome, displayed high electroporation efficiency and stable propagation of plasmids [11]. The mutant strain was further engineered and showed an 83 % increase of threonine production compared to the parental strain engineered with the same modifications [10]. l-threonine production was increased 2.4-fold when the corresponding gene modifications were introduced into *E. coli* strain MGF-01 that has a 22 % genome reduction [12]. For deletion mutants *E. coli* DGF-327 and DGF-298, an improvement of growth fitness was associated with the down-regulation of genes encoding chaperones and proteases [13]. A prophage-free *Corynebacterium glutamicum* with a 6 % reduced genome showed improved growth and fitness under SOS-response-inducing conditions, significantly increased transformation efficiency, as well as 30 % higher production of a heterologous model protein [14]. A genome-reduced *Streptomyces avermitilis* strain was also demonstrated to be highly suitable and effective for the heterologous production of antibiotics [8]. Additionally, a *Pseudomonas putida* strain with a reduced genome size was reported to achieve an increased biomass yield and enhanced heterologous gene expression [15].

However, deletion of some of the chosen non-essential regions (genes) also led to perturbations in the physiological characteristics of the strains, which led to a reduced growth rate and unstable phenotype. For *Bacillus subtilis* MG1M, with a genome reduction of 0.99 Mb, this meant a reduction in its growth rate, an aberrant cell morphology and unstable productivity of recombinant proteins during successive culture passages [16]. It was also reported that genome reduction decreased the carrying capacity of *E. coli* for foreign genes as well as its growth fitness [17]. Most recently, Unthan et al. investigated the impact of reductions encompassing 41 regions in the genome of *C. glutamicum* on the strain’s biological fitness. The single deletions of 10 individual regions resulted in impaired growth rates, and 26 regions were unnecessary for maintaining biological fitness at wild-type level [18].

*Bacillus subtilis* and related bacteria are widely used as hosts for industrial production of enzymes and other bio-based chemicals [19]. Some efforts have been made to streamline the genome of *B. subtilis* and improve its performance and suitability as a chassis cell. The *B. subtilis* ∆6 strain, which has a 7.7 % genome reduction, showed normal competence and sporulation phenotype, but exhibited a change of motility [20]. The strain MGB874, with about 20.7 % genome reduction, showed a reduced growth rate and aberrant gene transcription patterns. Moreover, the strain showed clearly improved characteristics compared to the wild-type concerning the production of alkaline cellulase and alkaline protease, displaying both a higher cell yield and a higher specific productivity [21, 22]. However, the production of the alkaline α-amylase AmyK38 was significantly decreased in strain MGB874 [23]. After introduction of an exogenous alkaline cellulase gene, the newly constructed cellulase-producing MGB874 exhibited a significantly increased flux in the pentose phosphate pathway, compared to an equivalently modified cellulase-producing derivative of the wild-type strain [24].

Apart from enzymes, *B. subtilis* has also been used widely for the production of small-molecule chemicals such as nucleosides [25], riboflavin [26], d-ribose [27], 2,3-butanediol [28] and acetoin [29]. Compared to other kinds of chemicals, the accumulation of nucleosides is more likely to be positively affected by genome reduction, since a part of the metabolic resources which would have been used for genome replication becomes available. Thus, we focused on the potential of genome reduction in *B. subtilis* for improving the production of guanosine and thymidine. We constructed genome-reduced strains of *B. subtilis* by reducing the genome size by up to 814.4 kb, and investigated the impacts of genome reduction on cell growth features, spore formation, transformation efficiency and maintenance energy requirements. Subsequently, by introducing genetic modifications which induce guanosine and thymidine accumulation, we demonstrated the great advantages and increased effectiveness of genome-reduced strains as chassis cells for the production of nucleoside products.

## Results and discussion

### Construction of genome-reduced *B. subtilis* strains

There are latent prophages, gene clusters related to antibiotic synthesis, and other regions scattered across the genome of *B. subtilis 168,* which do not contribute to performance in a typical industrial strain [20, 21, 30]. In order to avoid serious deviations of the terminus of replication from the original position due to concomitant deletion of multiple fragments, we carried out deletions of the non-essential regions in a pre-designed order (see Additional file 1: Figure S1). We sequentially deleted all known prophage regions (pro1, 2, 3, 4, 5, 6, spβ, *skin* and PBSX), antibiotic production operons (*pps*, *pks*) as well as a number of additional, miscellaneous non-essential regions (*ycxB*-*sipU*, *yisB*-*yitD*, *pdp*-*rocR*, *yrkS*-*yraK*, *yybP*-*yyaJ*, *ydeK*-*ydjC*, *lytH* –*yurT*, *sboA*-*ywhH*). The sizes of deleted regions added up to a total 814.4 kb. Although the regions chosen were somewhat similar to previous work reported by Morimoto et al. [21], a number of specific genes and the lengths of target regions deleted in this study were different (Additional file 1: Table S1). For example, some genes involved in spore development such as *spoIVCB, spoIIIC, spo0A* and *sigE* were not deleted in our study. On the other hand, we also eliminated some of the non-essential genes, which were not knocked out in the Morimoto et al. study, such as *ydhU*, *yitD*, *sspG* and *yurS*. Taken as a whole, we deleted a smaller number of non-essential regions (see Additional file 1: Table S1) than what the above-mentioned study reported [21].

Large-scale genome deletions are likely to affect cell growth, cell division, spore differentiation and substrate as well as energy utilization. However, owing to our limited understanding of genes still lacking annotation, as well as complex interactions of known genes, the effects of genome reduction on cell physiology are difficult to predict and have to be tested experimentally. Thus, in order to assess the impact of genome reduction on physiological characteristics and phenotypes, the strains BSK582, BSK665, BSK756, and BSK814 were selected for further study.

### Cell growth and substrate consumption

The growth curves of selected strains cultivated in M9 medium are shown in Additional file 1: Figure S2. Compared with the parental strain BSF1, the specific rates of cell growth and glucose consumption of the genome-reduced strains decreased gradually along with successively larger genome reductions. For strain BSK814, the maximal specific growth rate was only 0.41 ± 0.02 h^−1^ which constituted a decrease of 24 % compared to the parental strain (Table 1). The glucose consumption rate was 4.87 ± 0.25 mmol g (cdw)^−1^ h^−1^ which amounts to a decrease of about 25.6 %. The specific growth rate of BSK814 was almost the same as that of strain MGB874, the generation time of which was reported to be 100 min when the strain was cultured in Spizizen’s minimal medium [21]. Because many genes were deleted simultaneously as part of larger genome fragments, it is difficult to pinpoint individual genes that are most closely associated with the observed reduced growth rates. The results indicated that the successive deletion of additional genomic regions had a cumulative negative effect on cell growth and substrate consumption rate.

**Table 1:** Comparison of growth-related data of genome-reduced strains and the parental strain under aerobic conditions

Strains	BSF1	BSK582	BSK665	BSK756	BSK814
Specific growth rate (h^−1^)^a^	0.54 ± 0.03	0.48 ± 0.03	0.43 ± 0.02	0.42 ± 0.02	0.41 ± 0.02
CDW (g L^−1^)^b^	1.62 ± 0.02	1.64 ± 0.01	1.80 ± 0.03	2.17 ± 0.02	2.20 ± 0.03
Specific glucose uptake rate (mmol g^−1^cdw h^−1^)	6.55 ± 0.32	6.20 ± 0.26	5.30 ± 0.50	5.05 ± 0.31	4. 87 ± 0.25
Glucose consumption (g L^−1^)	6.56 ± 0.12	6.19 ± 0.10	6.77 ± 0.03	6.44 ± 0.10	6.38 ± 0. 16
Biomass yield (g/g glucose)	0.26 ± 0.01	0.28 ± 0.01	0.29 ± 0.02	0.35 ± 0.01	0.36 ± 0.01

Standard deviations were calculated from three biological replicates

^a^ Batch cultures were performed in 100 ml M9 medium with 1 % (w/v) glucose as described in method section

^b^ CDW is the final cell dry weight. At end of fermentation, cells were harvested and dried at 80 °C to a constant weight. Cell dry weight was determined by weighting the dried cells

However, the OD_600_ of these strains exhibited an opposite trend. All the genome-reduced strains reached a higher OD_600_ than the parental strain at the plateau stage of growth. One possibility is that changes in cell morphology might influence the OD_600_ measurements. However, based on microscopic observation (see Additional file 1: Figure S3), the cell size and shape of the strains in stationary phase did not exhibit obvious changes compared to the parental strain. Thus, the enhanced cell OD_600_ can be attributed to changes in cell physiology. The cell dry weigh (cdw) of strain BSK814 reached 2.2 g/L, which was 37 % higher than that of the parental strain. However, no obvious difference between the CDW of genome-reduced strains and the parental strain was observed when they were cultured in LB medium (data not shown). This observation was not consistent with results obtained in the study by Morimoto et al., which showed that the biomass values obtained with the genome reduced strain MGB874 and that of the wild type strain 168 were similar to each other in both LB and Spizizen’s minimal medium [21]. Due to lower glucose utilization rates and higher biomass, the biomass yields of the genome reduced strains also gradually increased with the size of genome reduction (Table 1). This observation might be related to the removal of genes encoding lysozymes such as CwlC and LytC, both of which play an important role in phage infection, cell division and differentiation [31, 32]. It was thus interesting to determine if there are any differences in cell autolysis between the genome-reduced strains and the parental strain.

### Analysis of cell autolysis behaviour

**Fig. 1:**
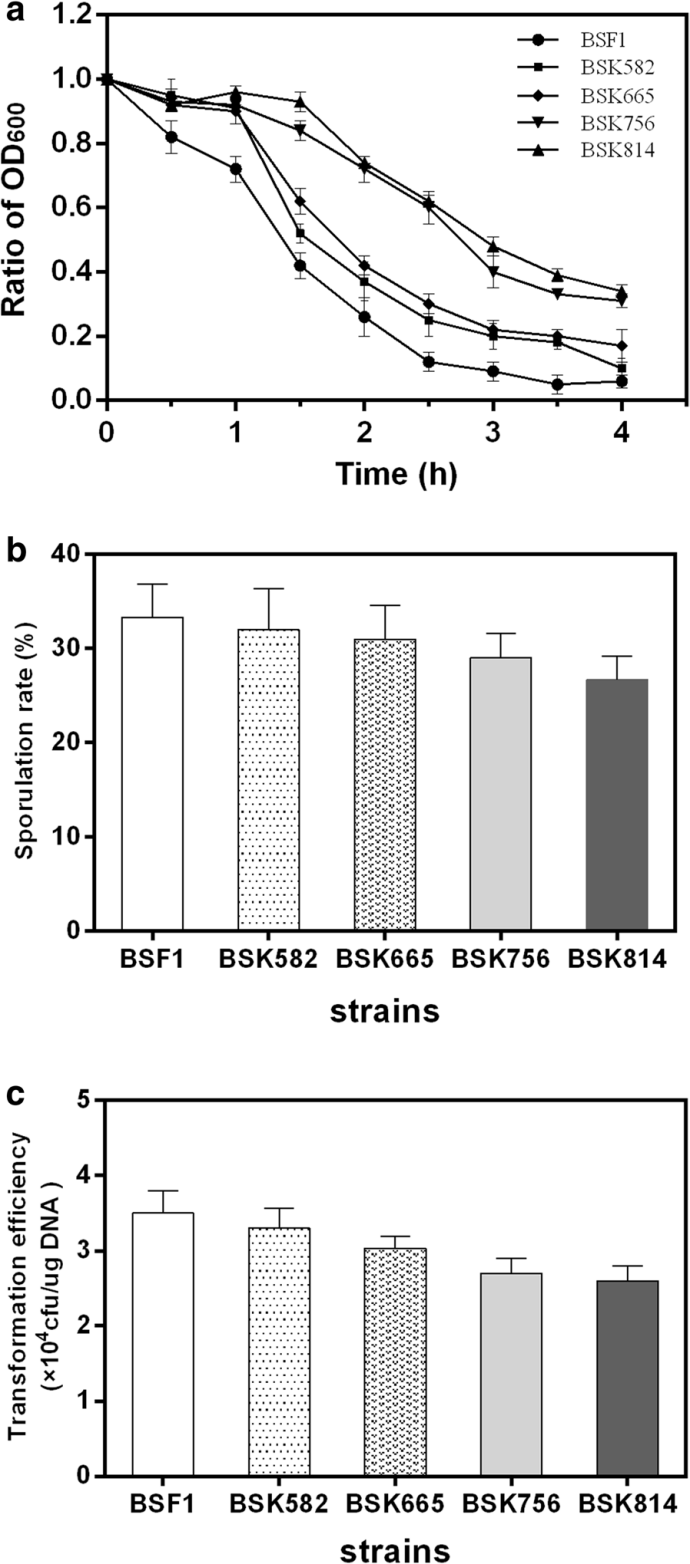
Cell autolysis, sporulation ratios and transformation efficiencies of genome-reduced strains and their parental strain. **a** Cell autolysis assay. Autolysis was represented by the ratio of the OD_600_ at the indicated time-points to the corresponding initial culture OD_600_. **b** Sporulation ratios. **c** Transformation efficiencies. All experiments were performed in triplicate

To investigate the effect of genome reduction on cell autolysis, we performed a cell lysis assay. As shown in Fig. 1a, all genome-reduced strains showed lower cell autolysis ratios compared to the parent strain BSF1. It has been reported that the deletion of the prophage and the lytic enzyme encoding gene *skfA*, which is located in the pro1 region, contributed to a decrease of cell lysis [33]. As an illustration, nearly 69 and 66 % of the BSK756 and BSK814 cells autolysed within 4 h, respectively, while 94 % of BSF1 cells autolysed within the same timeframe. There was also a significant difference between the autolysis ratios of BSK582 and BSK756 (83 vs 69 %). This might be caused by the deletion of regions such as PBSX and *skin* in BSK756. It was reported that deletion of *xpf,* which is located in the PBSX region, could also reduce the autolysis ratio [33]. On the other hand, the strain BSK814 showed an autolysis ratio and CDW similar to BSK756 (Fig. 1a; Table 1, respectively), which suggested that there were no additional active genes encoding potential autolytic lysozymes in the three deleted fragments, pro4, *lytH*-*yurT* and *sboA*-*ywhH*.

### Spore germination

Endospore development and germination are basic physiological characteristics of the *Bacillus* genus, and the process of sporulation can be initiated under conditions of nutrient limitation. To assess the impact of genome streamlining on spore development, we examined the sporulation ratios of the genome-reduced mutant strains. The sporulation ratio of strain BSK582 was nearly unchanged compared to the parental strain. However, as the genome reduction size increased stepwise from 581.9 to 814.4 kb, the corresponding sporulation ratios did exhibit a slight declining trend (from 33.5 to 26 %, Fig. 1b), although it was not statistically significant. This result can be attributed to the deletion of genes related to endospore development, including *cgeA, cgeE, cotP, rapF,gerkA, gerPA* and *gerPB*. The genes *cgeA* and *cgeE* are involved in the maturation of the outermost spore layer, whereas *cotP* encodes a spore-coat protein. It was deduced that a deletion of these genes could damage or inhibit the sporulation process [34]. In addition, deletion of genes *gerkA*, *gerPA* and *gerPB* also led to changes in the structure and composition of the endospores, which reduced their heat resistance [35, 36]. Morimoto et al. reported that the strain MGB874 did not form spores because some key sporulation genes were deleted, including *spoIVCB* and *spoIIIC* [21]. However, we did not remove genes *spoIVCB*, *spoIIIC*, *spo0A* or *sigE* in this study, so the sporulation characteristics of our genome-reduced strains were quite different from those of strain MGB874.

### Transformation efficiency

As ideal chassis cells, genome-reduced strains should maintain a high transformation efficiency to facilitate further genetic modifications. In order to understand the impact of genome reduction on transformation, we measured the transformation efficiencies of the four genome-reduced strains. As shown in Fig. 1c, the transformation efficiency gradually decreased as the genome reductions increased in size. The strain BSK814 exhibited a transformation efficiency of 2.8 × 10^4^ transformants per µg of plasmid DNA, which constitutes a decrease of 22 % compared to the parental strain. It has been reported that the transformation frequency of *B. subtilis* ∆6 was similar to that of the parental strain [20]. We also observed that the transformation efficiencies of the strains with genome reduction sizes of less than 581.9 kb were on par with the parental strain (data not shown). The decreased transformation efficiency might be attributed to deletion of genes involved in competence development. However, we did not find any genes which are known to be directly associated with cell competence in the deleted regions. Natural competence of *B. subtilis* can be induced when cells undergo starvation or environmental stress, which is controlled by the quorum sensing mechanism [37]. As for the genome-reduced strains, changes in physiological features and overall organization of the genome might affect the development of cell competence indirectly. In the study by Morimoto et al., it was found that the expression of *comK* and *degU*, which are involved in two-component signal transduction, together with a number of competence genes activated directly or indirectly by ComK, was induced earlier in MGB874 than the wild type strain, which resulted in earlier competence development. However, the impact of this change in competence development on the transformation efficiency of MGB874 was not mentioned in the report [21]. While the highest transformation efficiency for the parental strain BSF1 was obtained after 90 min of cultivation in SM2 medium, as reported by Vojcic [38], we observed slower growth in SM2 medium for BSK756 and BSK814, and the optimal transformation efficiencies were obtained when the cultivation time was 2.5 and 3.5 h, respectively. Transformation efficiency is a complex feature that is affected by many factors such as cell status, growth phase, nutrient availability, DNA uptake mechanisms, concentration and configuration of donor DNA and so on [39]. Notwithstanding these complex factors influencing transformation efficiency, our results indicate that the decreased transformation efficiency of the genome-reduced strains may be mainly a result of the change in growth phase due to their slower growth rates in SM2 medium.

### Maintenance coefficients

**Fig. 2:**
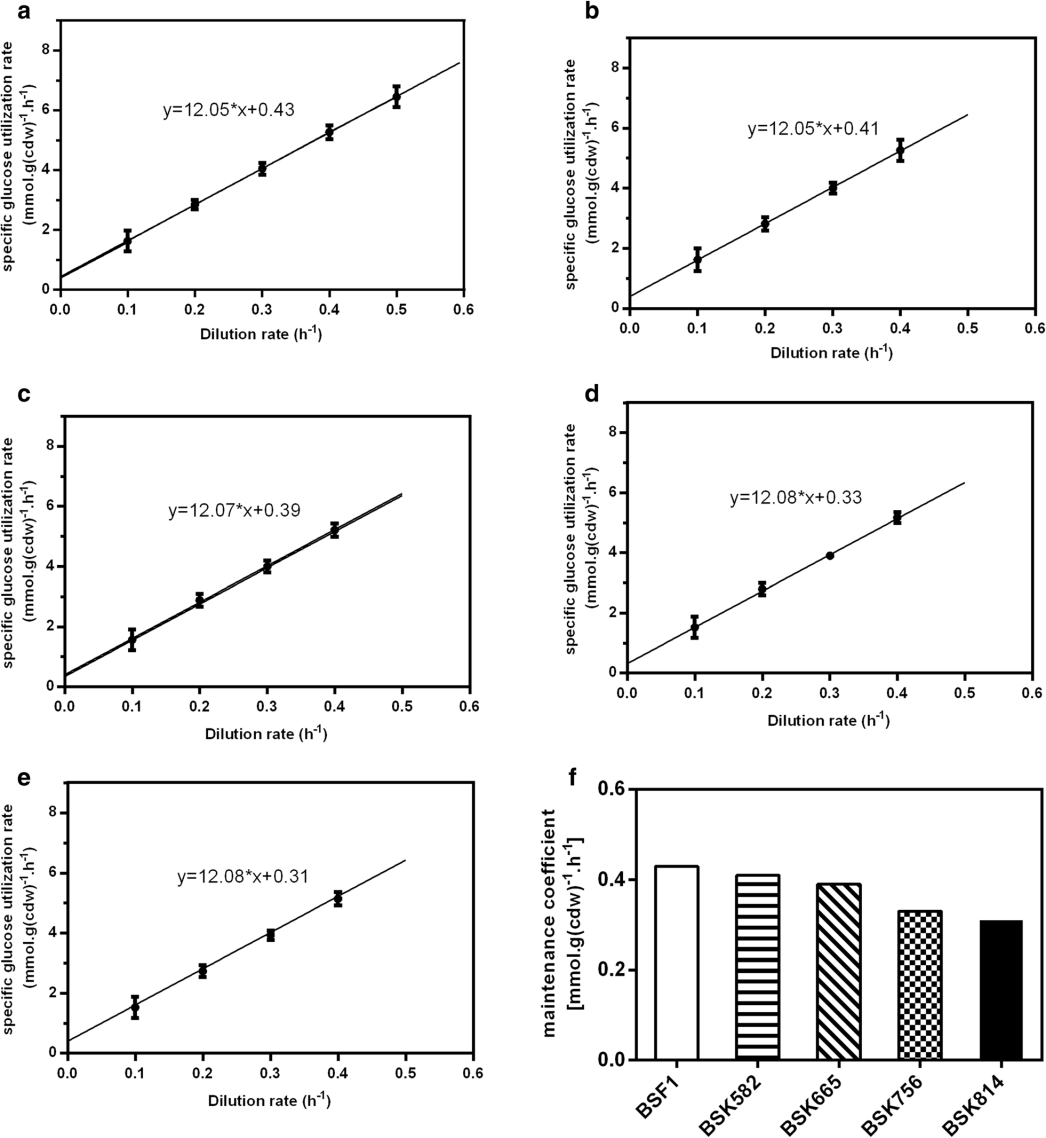
Specific glucose consumption rate as a function of dilution rate in glucose-limited chemostat cultures of the indicated strains. **a** The control strain BSF1. **b** BSK582. **c** BSK665. **d** BSK756. **e** BSK814. **f** Maintenance coefficient

The maintenance energy coefficient is a physiological parameter that reflects the energy required for maintaining cellular homeostasis [40]. Consequently, a low maintenance energy metabolism is generally an important selection criterion for an appropriate chassis organism in order to reduce non-productive substrate consumption during the bioprocess [1, 41]. To assess whether large-scale genome reduction has a significant effect on maintenance metabolism, we determined the substrate maintenance coefficients of the genome-reduced strains in glucose-limited chemostat cultures. As shown in Fig. 2, the substrate maintenance coefficients showed a decreasing trend with increased size of genome reductions. The control strain (*B. subtilis* 168 ∆*upp*::*neo*) had the highest maintenance coefficient of 0.43 mmol g (cdw)^−1^ h^−1^. This value is a bit higher than that of the wild-type strain 168, which was reported to be 0.39 mmol g (cdw)^−1^ h^−1^ [42]. The difference might be caused by the changed genotype (interruption of *upp* gene and expression of the marker gene, *neo*), as well as the different minimal media and culture conditions employed in the two studies. The strain BSK814, which has the largest deletion size of 814.4 kb, had the lowest maintenance coefficient of 0.31 mmol g (cdw)^−1^ h^−1^, which constitutes a decrease of 28 % compared to the control strain. Tännler et al. reported that a knockout of the gene *sigE* resulted in an obvious decrease in substrate maintenance coefficient [42]. However, *sigE* was not disrupted in our study, so the significant decrease in maintenance coefficient may be mainly attributed to the large-scale genome deletion itself. Recently, it has been reported that genome-reduced *Pseudomonas putida* strains also had lower maintenance demands and maintained a higher level of intracellular ATP than the wild-type strain under fast growth conditions [15]. Considering that the deletion of nonessential genes can make cells spend less energy and building blocks on cellular processes not associated with growth, a lower maintenance energy metabolism might be a common feature of genome-reduced strains. What’s more, the maximum biomass yields of all the genome-reduced strains were almost the same as that of control strain under continuous cultivation, and the value (about 0.46 g cdw/g glucose) was much higher than those measured in batch cultures (Table 1).

### Application of genome-reduced strains as chassis for guanosine and thymidine production

Guanosine is an important commercial additive used in the medicine and food industries, and thymidine is a commercially useful precursor in the chemical synthesis of various antiviral drugs [43]. Both nucleosides are precursors in the synthesis of nucleic acids. In order to explore if genome-reduced strains were better suited for the production these products, we constructed guanosine and thymidine producers by using these strains as chassis cells.

### Engineering genome-reduced strains as chassis for guanosine production

**Fig. 3:**
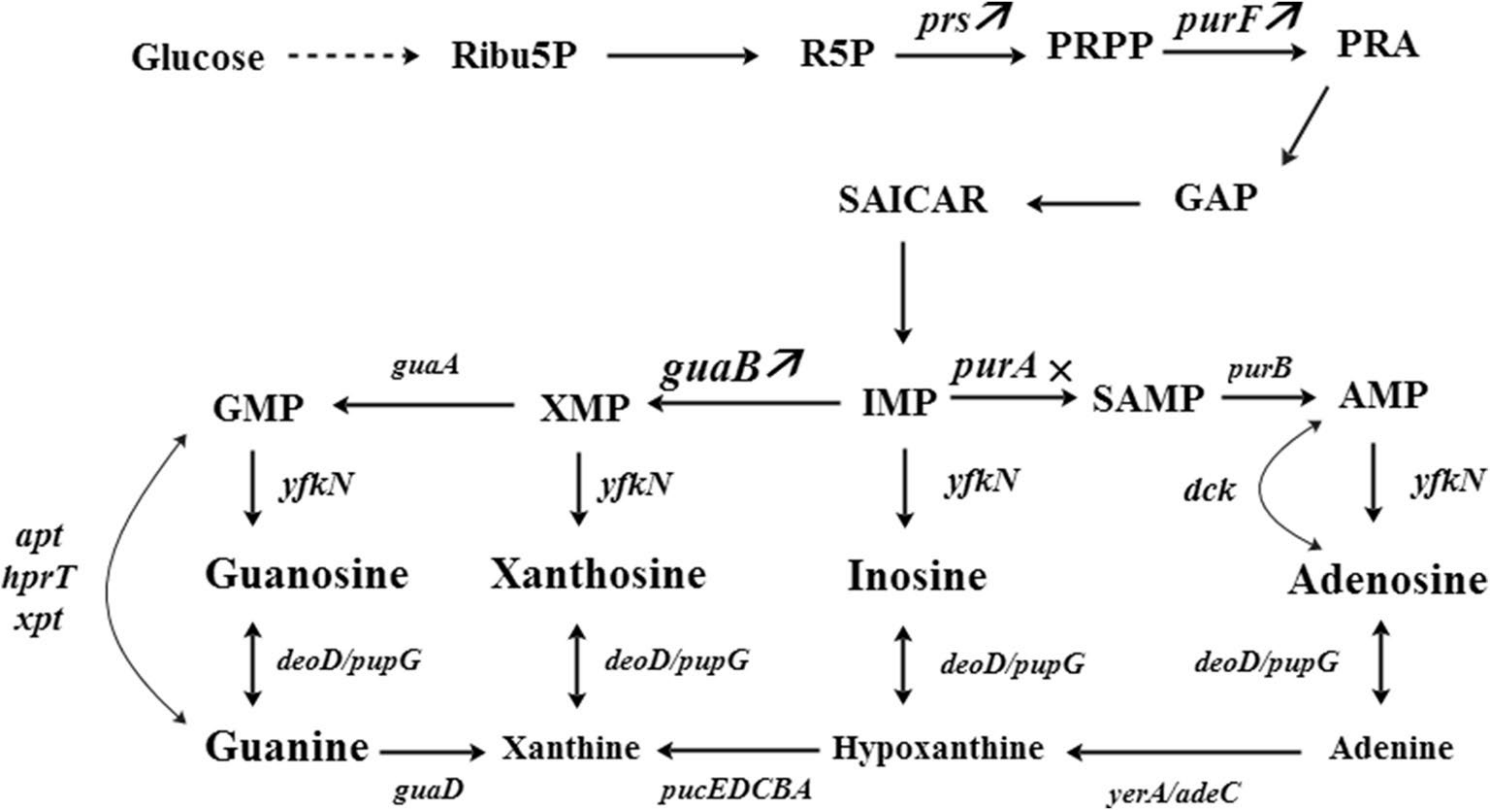
Engineering strategy for guanosine production. A deletion of the gene *purA* was combined with overexpression of the genes *prs, purF, and guaB* under the control of the strong P_43_ promoter. The gene abbreviations are as follows: *purA* encodes adenylosuccinate synthase; *prs* encodes ribose-phosphate pyrophosphokinase; *purF* encodes amidophosphoribosyltransferase; *guaB* encodes IMP dehydrogenase

**Fig. 4:**
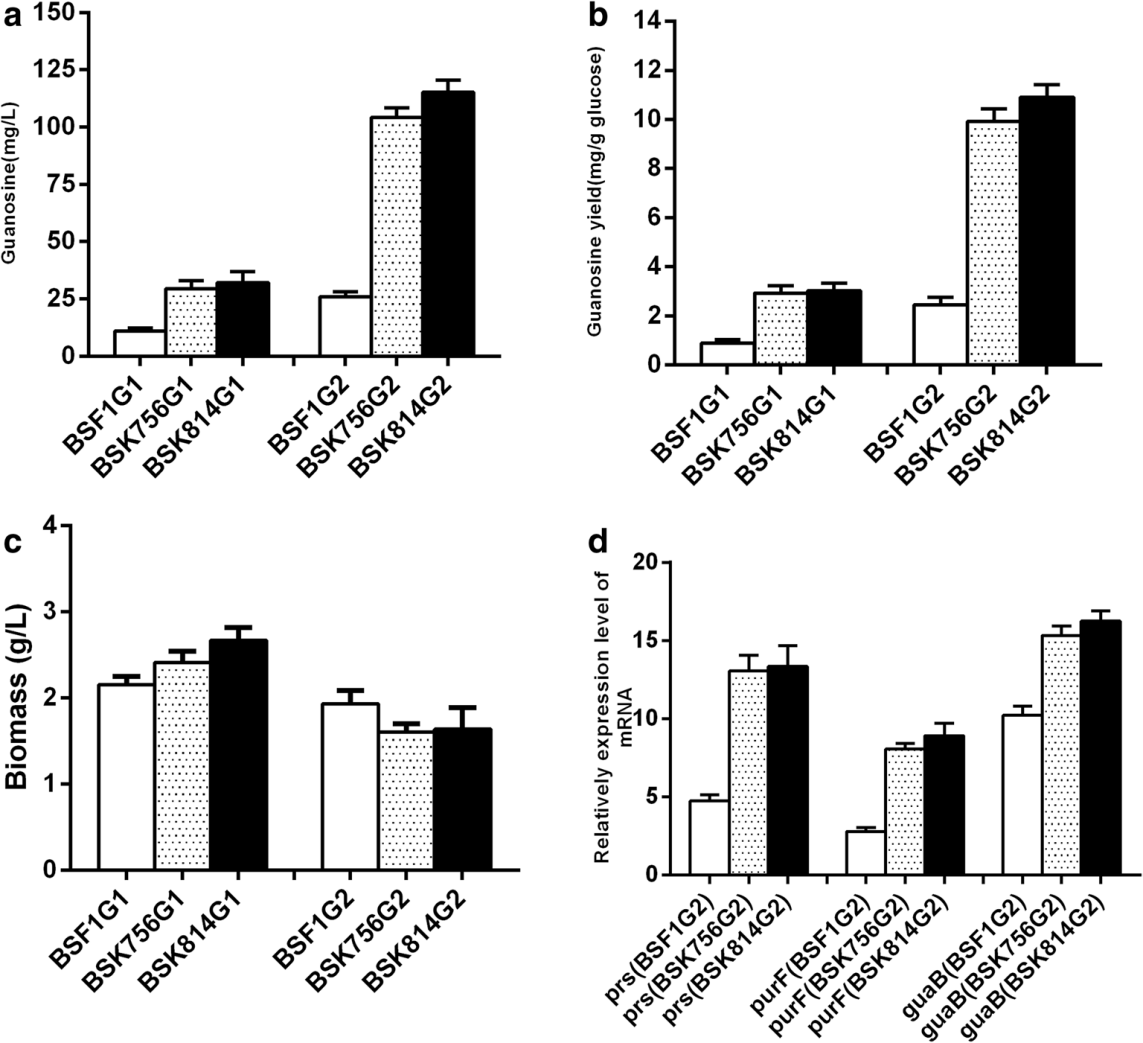
Relevant indicators of guanosine production by engineered *B. subtilis strains.* **a** Guanosine production. **b** Guanosine yield. **c** Biomass. **d** Transcriptional expression levels of relevant genes in strains BSK756G2, BSK814G2 and BSF1G2. The strains were cultivated in 100 ml LBG medium at 37 °C under constant orbital shaking at 220 rpm. Biomass yields were measured at 28 h

The strains BSK756 and BSK814, together with BSF1 as control, were engineered to produce guanosine. The corresponding genetic modification strategy is shown in Fig. 3. Firstly, we deleted *purA* to redirect carbon flux into the guanosine synthesis branch at the IMP node. The resulting stains BSF1G1, BSK756G1 and BSK814G1 were all auxotrophic for adenine, and produced 10.9, 29.5 and 32.2 mg/L of guanosine, respectively (Fig. 4a). Compared with the strain BSF1G1, the guanosine yields of BSK756G1 and BSK814G1 increased 2.7 and 2.9-fold, respectively (Fig. 4b). To further increase the flux from ribose-5-phosphate to GMP, we overexpressed the *prs, purF* and *guaB* genes by placing them on a plasmid under the control of the strong P_43_ promoter. To achieve this, the strains BSF1G1, BSK756G1 and BSK814G1 were transformed with the triple gene overexpression plasmid pHP13-ppg, which yielded the strains BSF1G2, BSK756G2 and BSK814G2. BSK756G2 and BSK814G2 produced 104.2 and 115.2 mg/L of guanosine in flask batch culture, which constituted a 4.0 and 4.4-fold increase over the guanosine produced by the control strain BSF1G2 (Fig. 4a). Additionally, the guanosine yields of BSK756G2 and BSK814G2 were also 4.0–4.4-fold higher than the yield of the strain BSF1G2 (Fig. 4b).

### Engineering genome-reduced strains as chassis for thymidine production

**Fig. 5:**
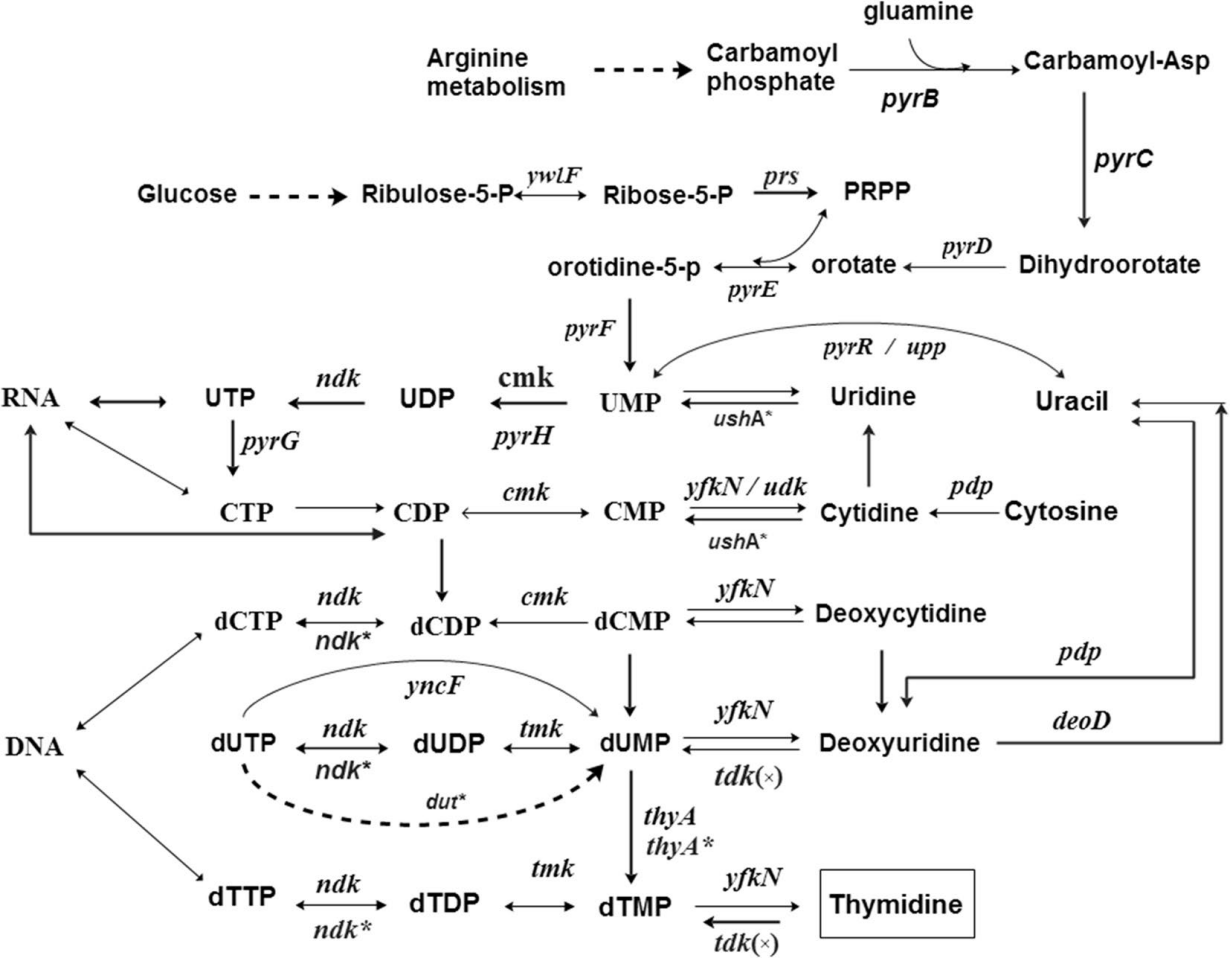
Engineering strategy for thymidine production. The gene *tdk* was deleted, and the gene *prs* was overexpressed by integrating a strong promoter via single crossover recombination; The genes *ushA*, *thyA*, *dut*, and *ndk* from *E. coli* were overexpressed from a plasmid under the control of the strong P43 promoter. The gene abbreviations are as follows: *tdk* encodes thymidine kinase; *ushA* encodes 5′-nucleotidase/UDP-sugar diphosphatase; *thyA* encodes thymidylate synthase; *dut* encodes dUTP pyrophosphatase; *ndk* encodes nucleoside-diphosphate kinase

**Fig. 6:**
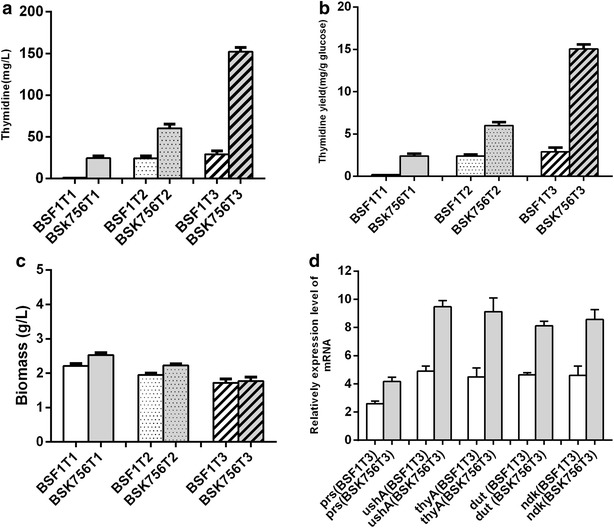
Relevant indicators of thymidine production by engineered *B. subtilis* strains. **a** Thymidine production. **b** Thymidine yield. **c** Biomass **d** Transcriptional expression levels of relevant genes in the strains BSK756T3 and BSF1T3. The strains were cultivated in 100 ml LBG medium at 37 °C under constant orbital shaking at 220 rpm. Biomass yields were measured after 30 h

For thymidine production, the strain BSK756 and the parental strain BSF1 were selected as the chassis strains, and the genetic modification strategy was conducted as shown in Fig. 5. We firstly knocked out the *tdk* gene, which encodes thymidine kinase (EC 2.7.1.21), in order to avoid degradation of thymidine. This modification yielded the strains BSF1T1 and BSK756T1, respectively. Interestingly, the strain BSF1T1 did not accumulate thymidine at all, while BSK756T1 produced 24.5 mg/L of thymidine in flask batch culture (Fig. 6a). In *E. coli,* disruption of all three of the genes *deoA*, *tdk* and *udp*, which are involved in the thymidine salvage pathway, could completely abolish product degradation, and even this triple knockout resulted in the production of only a small amount of thymidine [44]. However, in *B. subtilis,* we only found gene *tdk* (encodes thymidine kinase, EC 2.7.1.21) and *pdp* (encodes a pyrimidine-nucleoside phosphorylase, EC 2.4.2.2), which appear to share equivalent functions. Gene *pdp* shares similar functions to both *deoA* (thymidine phosphorylase, EC 2.4.2.2) and *udp* (uridine phosphorylase, EC 2.4.2.3) of *E. coli.* No gene in *B. subtilis* was annotated to encode uridine phosphorylase, which would be the equivalent of *udp* in *E. coli.* What’s more, gene *pdp* was deleted in BSK756 as part of the *pdp*-*rocR* region (Additional file 1: Table S1), and this might be the real reason why thymidine could be accumulated by BSK756T1 but not by BSF1T1. Next, we overexpressed the gene *prs* in BSF1T1 and BSK756T1 from the chromosome, by inserting a P_43_ promoter and *prs* gene( P_43_-*prs*) at the *bdhA* locus in the genome. This modification yielded the strains BSF1T2 and BSK756T2, respectively. The latter strain produced 60.3 mg/L thymidine, which is approximately 2.5-fold more than the production of the former. Finally, the two strains were further transformed with plasmid pHP13-untd, in which the *ushA*, *thyA, dut* and *ndk* genes from *E. coli* were overexpressed under the control of the strong P_43_ promoter, which yielded the strains BSF1T3 and BSK756T3. In shake-flask cultivation, the thymidine production by BSK756T3 further increased to 151.2 mg/L, which was 5.2-fold higher than that of BSF1T3 (Fig. 6a), whereas the thymidine yield of BSK756T3 increased 5.0-fold compared to BSF1T3 (Fig. 6b).

### Changes in expression levels of genes involved in guanosine and thymidine production

When genome-reduced strains BSK756 and BSK814 were used as chassis cells for guanosine and thymidine production, all the derived engineered strains exhibited much higher productivities and yields than the corresponding control strains. One explanation for this result may be that the genome-reduced strains grew to a higher cell density. The biomass values of BSK756G1 and BSK814G1 were respectively 12.2 and 12.4 % higher than those of the control strain BSF1G1 (Fig. 4c). Similarly, thymidine-producing strains with genome reductions also showed increased biomass values to different extents, compared to the control strains (Fig. 6c). However, we observed an opposite phenomenon for strains BSK756G2 and BSK814G2, which did not support this tentative explanation.

The second possible reason may be that the deletion of unnecessary regions from the genome could lead to more efficient cellular metabolism. Lee et al. reported that most of the genes involved in central metabolism as well as l-threonine biosynthesis were up-regulated in the genome reduced *E.coli* MDS-205 [10]. A different line of research by Manabe et al. indicated that the transcriptional level of the gene *egl*-*237,* which encodes an extracellular alkaline cellulase, was significantly higher in strain MGB874 than in wild type 168, which directly resulted in a higher specific enzyme productivity in the genome-reduced strain [22]. To verify this, we measured the transcriptional levels of all the overexpressed genes related to guanosine and thymidine production. As shown in Fig. 4d, the transcriptional levels of the *prs, purF* and *guaB* genes in the two genome-reduced strains BSK756G2 and BSK814G2 were almost identical, but were respectively 2.7, 2.9 and 1.5-fold higher than those in BSF1G2. For genes *prs*, *ushA*, *thyA, dut* and *ndk*, the transcription levels in BSK756T3 were respectively 1.36, 1.92 1.81, 1.67 and 1.74-fold higher compared with those in BSF1T3 (Fig. 6d). Therefore, our results also indicated that genome-reduced strains might have a more efficient overall cellular metabolism, which might indirectly increase transcription efficiency. In addition, the four genes *ushA*, *thyA, dut* and *ndk* on the plasmid pHP13-untd showed a markedly higher degree of increase in their respective transcription levels than the *prs* gene, which was integrated into the chromosome. This could be the result of the increased copy numbers of plasmids propagated in genome-reduced strains, which is a peculiar phenomenon demonstrated earlier by Manabe et al. [22]. Ultimately, this observation might be caused mainly by the lower substrate maintenance coefficients caused by genome reduction, which allows the strains to spend more ATP on plasmid replication, in addition to increased product biosynthesis and cell growth.

Finally, since the need for direct precursors and building blocks of nucleic acids decreases directly due to large-scale genome reduction, this type of genetic manipulation makes it easier for the chassis cells to accumulate these building blocks. From this point of view, we also expect the genome-reduced strains constructed as part of this work to show greatly improved characteristics over the wild-type strain for production of other nucleosides and derived chemicals, and this is an aspect which will be further explored in our future research.

## Conclusions

In the present study, nonessential regions in the genome of *B. subtilis* 168 were deleted stepwise, whereby the maximum size of genome reduction reached 814.4 kb. Four strains with respective genome deletion sizes of 581.9, 665.2, 756.8 and 814.4 kb were selected to investigate the effects of genome reduction on the strains’ characteristics. The specific glucose utilization and growth rates, sporulation ratios, transformation efficiencies and maintenance coefficients gradually decreased along with increasing sizes of genome reduction. On the other hand, the cell yields exhibited an opposite trend. We further constructed guanosine and thymidine producing strains by using the genome reduced strains BSK756 and/or BSK814 as chassis cells. In flask cultivation, the guanosine-producing strain BSK814G2 and thymidine-producing strain BSK756T3 accumulated 115.2 mg/L of guanosine and 151.2 mg/L thymidine, which represented a 4.4 and 5.2-fold increase compared to full-genome control strains, respectively. Although the product yields are relatively low and additional genetic modifications are needed to further improve the strains, the results presented here clearly demonstrate that strains with large-scale genome reduction possess greatly improved characteristics as chassis cells for the production of nucleoside products.

## Methods

### Strains, plasmids and media

All strains and plasmids used in this study are summarized in Tables 2 and 3. The strain BSF1 (*B. subtilis* 168 *∆upp::neo*) was selected as the parental strain for genome reduction. *Escherichia coli* DH5α was used for construction and propagation of plasmids. *Escherichia coli* and *B. subtilis* cells were cultured at 37 °C in LB medium supplemented with appropriate antibiotics. Mutant selection was conducted in MM medium containing 2.0 g/L (NH_4_)_2_SO_4_ 6.0 g/L KH_2_PO_4_, 14.0 g/L K_2_HPO_4_, 1.2 g/L sodium citrate tribasic dihydrate (Na_3_C_6_H_5_O_7_.2H_2_O), 0.2 g/L MgSO_4_.7H_2_O, 8.0 g/L glucose, 50 mg/L tryptophan, 2 g/L glutamine, 10 μmol/L 5-fluorouracil (5FU), and trace element solution as published previously [45]. M9 medium, supplemented with 1 % (w/v) glucose, 100 mM MOPS and 50 mg/L tryptophan, was used for assessing growth of genome-reduced strains [46]. SM1 and SM2 media were used for the preparation of *B. subtilis* competent cells as described previously [38]. Glucose-limited medium for continuous culture contained components as reported by Sauer et al. [40]. LBG medium for guanosine and thymidine production contained 10 g/L tryptone, 5 g/L yeast extract, 10 g/L sodium chloride, 10 g/L water-free glucose and 0.1 mol/L MOPS. Where appropriate, 5 µg/ml chloramphenicol (Cm), 10 µg/ml kanamycin (Km) or 5 µg/ml erythromycin (Em) was added to promote plasmid retention. Unless otherwise specified, the reagents and antibiotics used in this study were purchased from Sangon biotech Co., Ltd (Shanghai, China) and were of the highest available purity.

**Table 2:** Strains used in this study

Strain	Genotype	Source
*E. coli* DH5α	Cloning host	Invitrogen
BSF1	*B.subtilis 168*Δ*upp::neo*	[28]
BSK582	BSF1 Δpro1 Δpro2 Δpro3 Δpro5 Δpro6 Δspβ Δpps Δ*pks* Δ(*ycxB*-*sipU*) Δ(*yisB*-*yitD*) Δ(*pdp*-*rocR*), 581.9 kb reduction of genome	This study
BSK665	BSK582 ΔSpβ’ Δ*skin* Δ(*yybP*-*yyaJ*), 665.2 kb reduction of genome	This study
BSK756	BSK665 ΔPBSX Δ(*ydeK*-*ydjC*), 756.8 kb reduction of genome	This study
BSK814	BSK756 Δpro4 Δ(*lytH* -*yurT*) Δ(*sboA*-*ywhH*), 814.4 kb reduction of genome	This study
BSF1T1	BSF1 Δ*tdk*	This study
BSF1T2	BSF1 Δ*tdk* Δ*bdhA:*: P_43_-*prs*	This study
BSF1T3	BSF1 Δ*tdk* Δ*bdhA*::P_43_-*prs*, containing pHP13-untd	This study
BSK756T1	BSK756 Δ*tdk*	This study
BSK756T2	BSK756 Δ*tdk* Δ*bdhA:*: P_43_-*prs*	This study
BSK756T3	BSK756 Δ*tdk* Δ*bdhA*::P_43_-*prs,* containing pHP13-untd	This study
BSF1G1	BSF1 Δ*purA*	This study
BSF1G2	BSF1 Δ*purA*, containing pHP13-ppg	This study
BSK756G1	BSK756 Δ*purA*	This study
BSK756G2	BSK756 Δ*purA*, containing pHP13-ppg	This study
BSK814G1	BSK814 Δ*purA*	This study
BSK814G2	BSK814 Δ*purA*, containing pHP13-ppg	This study

**Table 3:** plasmids used in this study

plasmid	Genotype	Source
pU	pUC18, Amp^R^, containing *cat*-*upp* and two MCS regions	Lab stock
pCU	pUC18, Amp^R^, containing *cat*-*upp and a* MCS region	Lab stock
pU-pro1	pU, containing pro1 upstream, downstream flanks and an internal fragment in target region	This study
pU-pro2	pU, containing pro2 upstream, downstream flanks and an internal fragment in target region	This study
pCU-pro3	pCU, containing pro3 upstream and downstream flanks	This study
pU-pro4	pU, containing pro4 upstream, downstream flanks and an internal fragment in target region	This study
pU-pro5	pU, containing pro5 upstream, downstream flanks and an internal fragment in target region	This study
pU-pro6	pU, containing pro6 upstream, downstream flanks and an internal fragment in target region	This study
pU-PBSX	pU, containing upstream, downstream flanks of PBSX and an internal fragment in target region	This study
pU-*skin*	pU, containing upstream, downstream flanks of *skin* and an internal fragment in target region	This study
pU-spβ	pU, containing upstream, downstream flanks of spβ and an internal fragment in target region	This study
pU-spβ’	pU, containing upstream, downstream flanks of target region and an internal fragment in target region	This study
pU-yrkS-yraK	pU, containing upstream, downstream flanks of target and an internal fragment in target region	This study
pU-yisB-yitD	pU, containing upstream, downstream flanks of target and an internal fragment in target region	This study
pU-ycxB-sipU	pU, containing upstream, downstream flanks of target and an internal fragment in target region	This study
pU-pdp-rocR	pU, containing upstream, downstream flanks of target and an internal fragment in target region	This study
pCU-tdk	Amp^R^, Cm^R^, containing *tdk* upstream and downstream flanks of target region	This study
pCU-purA	Amp^R^, Cm^R^, containing *purA* upstream and downstream flanks	This study
pCU-bdhA-prs	Amp^R^, Cm^R^, containing *bdhA* upstream and downstream flanks, P_43-_ *prs*	This study
pHP13	Cm^R^, Em^R^, *B.subtils*-*E. coli* shuttle plasmid	Lab stock
pHP13-ppg	pHP13 replication, Cm^R^, Em^R^, P_43_-*prs*-*purF*-*guaB*	This study
pHP13-untd	pHP13 replication, Cm^R^, Em^R^, P_43_-*ushA*-*ndk*-*thyA*-*dut*	This study

Amp^R^, ampicillin resistance; Cm^R^, chloramphenicol resistance; Em^R^, erythromycin resistance

### Plasmid construction

The plasmids pU and pCU were used as backbones to construct targeting plasmids for genome streamlining. Upstream and downstream sequences of the target region or gene were amplified using PCR and assembled using splicing by overlap extension PCR (SOE-PCR), and cloned into the multiple cloning sites (MCS) of the backbone plasmids, yielding the plasmids pCU-tdk and pCU-purA. To improve the efficiency of large-scale deletions, a subset of the plasmids was constructed using the following method: the upstream sequence, downstream sequences of the target region as well as an internal sequence in the target region were amplified by PCR. The upstream sequence and the downstream sequence of the target region were fused by SOE-PCR, the resulting fragment was digested and ligated into the MCS-I site of plasmid pU. The internal sequence was cloned into the MCS-II site of plasmid pU. The derived plasmid, containing upstream sequence, internal sequence, *cat*-*upp* cassette, and downstream sequence, was linearized and used for deletions of large regions. For plasmid pHP13-ppg, the genes *prs*, *purF* and *guaB* were amplified from the genome of *B. subtilis* 168, the corresponding DNA fragments digested and ligated step by step into the plasmid pHP13, finally yielding the plasmid pHP13–ppg in which the genes *prs*, *purF* and *guaB* were overexpressed under the control of the strong P_43_ promoter. The plasmid pHP13-untd was constructed in the same way as described above. The genes *ushA*, *thyA, dut* and *ndk* were amplified from *E. coli* MG1655 genomic DNA by PCR. These corresponding DNA fragments were digested and ligated into the plasmid pHP13 step by step, generating plasmid pHP13-untd in which the transcription of all four genes was controlled by the strong P_43_ promoter. The plasmid pCU-bdhA-prs was constructed as reported previously [47].

### Genetic manipulation for genome reduction

To facilitate successive deletion procedures, we adopted a scarless deletion method using *upp* as counterselection marker (Additional file 1: Figure S4) [48]. A linearized targeting plasmid, containing homologous fragments flanking the counterselection cassette, was used to transform competent cells of the recipient strain. Positive transformants were selected on plates containing chloramphenicol and transferred into LB liquid medium. After cultivation for either 6 or 12 h, appropriate amounts of cells were spread onto MM agar plates. Colonies that grew on MM agar plates were further verified by PCR.

### Physiological analyses

Growth characterization of deletion mutants was performed by streaking the strains on LB agar plates from −80 °C glycerol stocks and cultivating at 37 °C, transferring single colonies from the plates into 50 ml M9 medium and culturing overnight at 37 °C under constant orbital shaking at 220 rpm. Subsequently 2 ml culture aliquots were used to inoculate 500 ml shake flasks containing 100 ml M9 medium and the cells further cultivated at 37 °C and 240 rpm. All experiments were performed in triplicate.

The cell lysis assay was performed using sodium azide as described previously [33]. Sodium azide is a cytochrome oxidase inhibitor and can lead to disruption of cell division. It is often used to monitor dynamic process of autolysis in real time. Briefly, the cells were grown in LB medium until mid-exponential phase, after which a final concentration of 0.05 M sodium azide was added to the culture medium and incubation continued at 37 °C and 220 rpm. The optical density (OD_600_) of the cultures was monitored periodically. Cell lysis was calculated by comparing the ratio of OD_600_ at each sampling time-point to the initial OD_600_.

To study spore formation of mutants, cells were incubated in Difco sporulation medium (DSM) and shaken at 37 °C for 24 h to induce sporulation. One millilitre aliquots of the DSM cultures were heated at 80 °C for 20 min to kill vegetative cells. Heated and unheated aliquots were diluted and plated on LB-agar plates. After incubation at 37 °C for 72 h, the ratio of the number of colonies from corresponding heated and unprocessed aliquots was used to calculate sporulation rates. All experiments were performed in triplicate.

Transformation efficiency was determined according to the method described by Vojcic et al. [38], with minor modifications. Briefly, fresh single colonies were inoculated into flasks containing SM1 medium. The resulting overnight cultures were then diluted with SM1 medium, the optical density at 600 nm adjusted to 0.3 in a volume of 10 ml and incubated at 37 °C and 220 rpm. When the cells exited the exponential growth phase, the cultures were transferred to 5 ml SM2 medium at a ratio of 1:1, and incubated until the cells reached the late exponential growth phase. The competent cells were contacted with 100 ng of pHP13 plasmid DNA. After incubation for 1 h, the cells were plated on selective medium plates and the numbers of the resulting colonies were used to calculate transformation efficiency values of the mutant strains.

To determine the maintenance coefficients of the strains, continuous cultivations of each strain were conducted in a 1.5-L Fermenter (BioFlo 110, New Brunswick Scientific Co. Inc.,USA) containing 0.4 L of glucose-limited medium. Seed cultures grown at 37 °C in 250 ml flasks containing 50 ml of seed medium were used to inoculate the fermenter. When glucose was nearly exhausted, the cultivation was switched from batch to chemostat mode. The fermentation volume was kept constant using an automated pump. The pH of the culture medium was maintained at 6.65 with 0.1 mol/L NaOH. Aeration rate, agitation speed and temperature were controlled at 0.2 vvm, 400 rpm and 37 °C, respectively. The dilution rate (D) was increased stepwise from 0.1 to 0.5 h^−1^. Each D value was determined by feeding medium at a predesigned flow rate. Cell growth subsequently reached a steady state at which cell density and glucose consumption rate remained unchanged for prolonged periods of time. Samples were collected and weighed after drying at 80 °C to constant weight, and glucose consumption was constantly monitored at the same time. Experimental values for the maintenance coefficient which were expressed as glucose consumption rate (m_glc_) were derived from fitted linear regression lines.

### Engineering guanosine and thymidine synthesis pathways

The strains BSK756 and BSK814 were selected for guanosine biosynthesis engineering. Plasmid pCU-purA was used to delete *purA* gene in the strains BSF1, BSK756 and BSK814, yielding the strains BSF1G1, BSK756G1 and BSK814G1, respectively. Subsequently, the plasmid pHP13-ppg was introduced into the *purA*-deficient mutants to overexpress genes *prs*, *purF* and *guaB*, resulting in strains BSF1G2, BSK756G2 and BSK814G2, respectively.

For thymidine production, *tdk* gene was deleted, and genes *prs*, *ushA*, *thyA, dut* and *ndk* were overexpressed. Firstly, *tdk* was knocked out using the plasmid pCU-tdk as described previously, resulting in strains BSF1T1 and BSK756T1. Subsequently, the plasmid pCU-bdhA-prs was introduced and integrated into the chromosome of the recipient cells by a first single-crossover event, and a second single-crossover event during 12 h cultivation in LB liquid medium yielded the mutant strains BSF1T2 and BSK756T2, in which *bdhA* has been replaced by the P_43_-prs fragment. Genes *ushA*, *thyA, dut* and *ndk* were overexpressed by introducing the plasmid pHP13-untd into BSF1T2 and BSK756T2, yielding BSF1T3 and BSK756T3, respectively. For fermentations, single colonies of *B. subtilis* were transferred into 5 ml LB medium and incubated at 37 °C and 220 rpm. The resulting overnight cultures were used to inoculate 500-ml shake flasks containing 100 ml LBG medium and cultivated at 37 °C and 220 rpm.

### Analytical methods

The shape and size of cell were examined and recorded using an Olympus CX41 microscope equipped with an digital camera (Olympus, Tokyo, Japan). All photomicrographs were taken at 1000× magnification. Cell growth was monitored by measuring the optical density at 600 nm (OD_600_) using a UV–Vis spectrophotometer(TU-1801, Beijing PuxiUniversal Co., Ltd., Beijing, China). Glucose consumption was quantified using a biosensor (SBA-40C, Biology Institute of Shandong Academy of Science, Shandong, China) [49]. Analysis of thymidine and guanosine concentrations was performed using reverse-phase HPLC (HP1100, Agilent, USA) equipped with a Luna C18 column (2)(150 × 4.6 mm, 5 µm, Phenomenex, USA) and the eluents were followed photometrically at 260 nm. For thymidine, the mobile phase was 4 % (v/v) acetonitrile/water containing 0.05 % (v/v) trifluoroacetic acid; The flow rate was 1 ml/min; The retention time for thymidine was 7.35 min [50]. For guanosine, the mobile phase was 4 % (v/v) acetonitrile/water; The flow rate was 0.6 ml/min; The retention time for guanosine was 13.21 min.

Quantitative real-time reverse transcription PCR analysis was performed as follows: cultures were harvested when cells reached the mid-exponential growth phase. RNA was extracted using the RNAprep pure Cell/Bacteria Kit (Tiangen, Beijing, China) according to the manufacturer’s protocol. Subsequently the RNA was used as template to amplify cDNA using the FastQuant RT Kit (Tiangen, Beijing, China). The resulting cDNA was analysed using a Light Cycler 480 II (Roche, Basel, Switzerland) and RealMasterMix (SYBR Green I, Tiangen, Beijing, China). The 16s rRNA gene *rrnA*-*16S* was used as internal control. Data were quantified using the 2^−ΔΔCt^ method.

## Additional file

10.1186/s12934-016-0494-7 Strategy for the construction of genome reduced strains of *B.subtilis*; **Figure S2.** Growth and glucose consumption of genome-reduced strains and the parental strain BSF1 in M9 medium; **Figure S3**. Cell morphology of genome-reduced strains and their parental strain BSF1; **Figure S4.** Schematic representation of the scarless deletion methods for genome streamlining; **Table S1.** Regions deleted in the course of genome reduction; **Table S2.** Primers used in this study.

## Abbreviations

5FU: 5-fluorouracil
Cm: chloramphenicol
Km: kanamycin
Em: erythromycin
DSM: difco sporulation medium
cdw: cell dry weigh
D: dilution rate
