# Supplementary material for: Characterization of genome-reduced Bacillus subtilis strains and their application for the production of guanosine and thymidine
Source: Microb Cell Fact. 2016 Jun 3;15:94. doi: 10.1186/s12934-016-0494-7 (PMC4893254; doi:10.1186/s12934-016-0494-7)
Supplement: Supplementary file 1 — 10.1186/s12934-016-0494-7 Strategy for the construction of genome reduced strains of B.subtilis; Figure S2. Growth and glucose consumption of genome-reduced strains and the parental strain BSF1 in M9 medium; Figure S3. Cell morphology of genome-reduced strains and their parental strain BSF1; Figure S4. Schematic representation of the scarless deletion methods for genome streamlining; Table S1. Regions deleted in the course of genome reduction; Table S2. Primers used in this study. [file 12934_2016_494_MOESM1_ESM.docx]

Microbial Cell Factories

SUPPLEMENTARY MATERIALS FOR THE PUBLICATION:

**Characterization of genome-reduced *Bacillus subtilis* strains and their application for the production of guanosine and thymidine**

**Authors:**

Yang Li^1,4^, Xujun Zhu^1^, Xueyu Zhang^1,3^, Jing Fu^1^, Zhiwen Wang^1^, Tao Chen^*1,2^ and Xueming Zhao^1^

^1^Key Laboratory of Systems Bioengineering (Ministry of Education); SynBio Research Platform, Collaborative Innovation Center of Chemical Science and Engineering (Tianjin), School of Chemical Engineering and Technology, Tianjin University, Tianjin, 300072, China

^2^Hubei Provincial Cooperative Innovation Center of Industrial Fermentation; Key Laboratory of Fermentation Engineering (Ministry of Education), Hubei University of Technology, Wuhan 430068, China

^3^Tianjin Vocational College of Bioengineering, Tianjin, 300462, China

^4^College of Life Science, Shihezi University, Shihezi 832000, China

^*^ Correspondence: [chentao@tju.edu.cn](mailto:chentao@tju.edu.cn)

**Supplementary figures:**


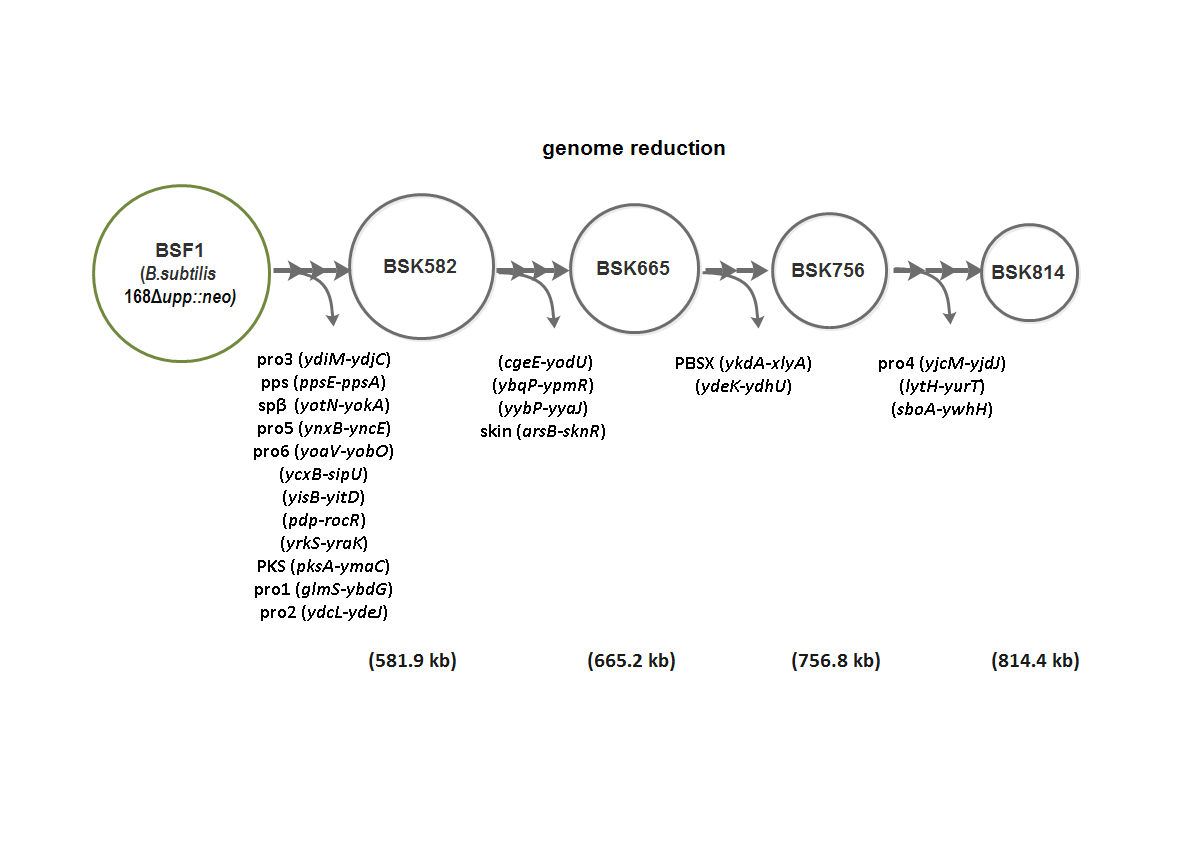


# Figure S1 Strategy for the construction of genome reduced strains of *B .subtilis*

The train BSF1, as initial strain, was used for genome reduction. All genome reduction manipulations were performed in a specific order. Non-essential regions, including pro3, pps, spβ, pro5, *ycxB-sipU*, *yisB-yitD*, *pdp-rocR*, *yrkS-yraK*, *pks*, pro1, and pro2 were deleted in the sequence listed, resulting in the strain BSK582 in which the total sizes of deleted regions added up to 581.94 kb. The regions *cgeE-yodU*, *ybqP-ypmR*, *yybP-yyaJ, and skin* were further deleted in that order, yielding *s*train BSK665, in which the sizes of deleted regions added up to 665.2 kb. PBSX *and ydeK-ydhU* were further deleted in strain BSK665, resulting in strain BSK756, in which the sizes of deleted regions added up to 756.8 kb. Finally, regions including pro4, *lytH –yurt*, *sboA-ywhH,* were further deleted in strain BSK756 and strain BSK814 was obtained in which the sizes of deleted regions added up to 814.4 kb.





# Figure S2 Growth and glucose consumption of genome-reduced strains and the parental strain BSF1 in M9 medium.

(a) Cell growth. (b) Glucose consumption. The strains were cultivated in 100 ml M9 medium with 1% (w/v) glucose at 37°C under constant orbital shaking at 240 rpm. OD600 and residual glucose were determined in 1 h intervals. The specific growth rate was calculated from five data points selected during the exponential phase by determining the slope of the linear regression curve in an lnOD600-h graph. The experiments were performed in three biological replicates.


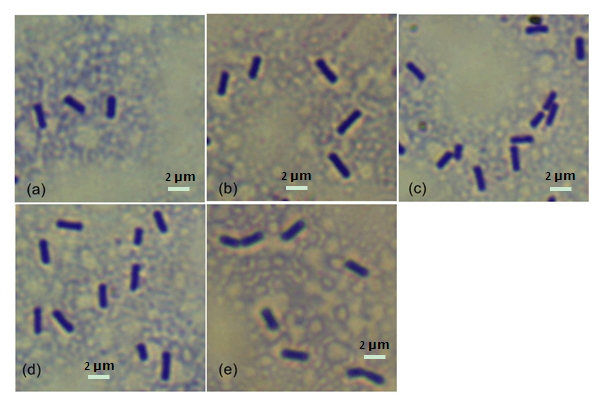


# Figure S3 Cell morphology of genome-reduced strains and their parental strain BSF1.

1. strain BSF1; (b) strain BSK582; (c) strain BSK665; (d) strain BSK756; (e) strain BSK814. All strains were cultivated in M9 medium until cells entered the stationary phase. Images were captured using Olympus CX41 light microscope (Olympus, Tokyo, Japan) equipped with digital camera, at 1,000× magnification. The scale bar represents 2 μm.

**
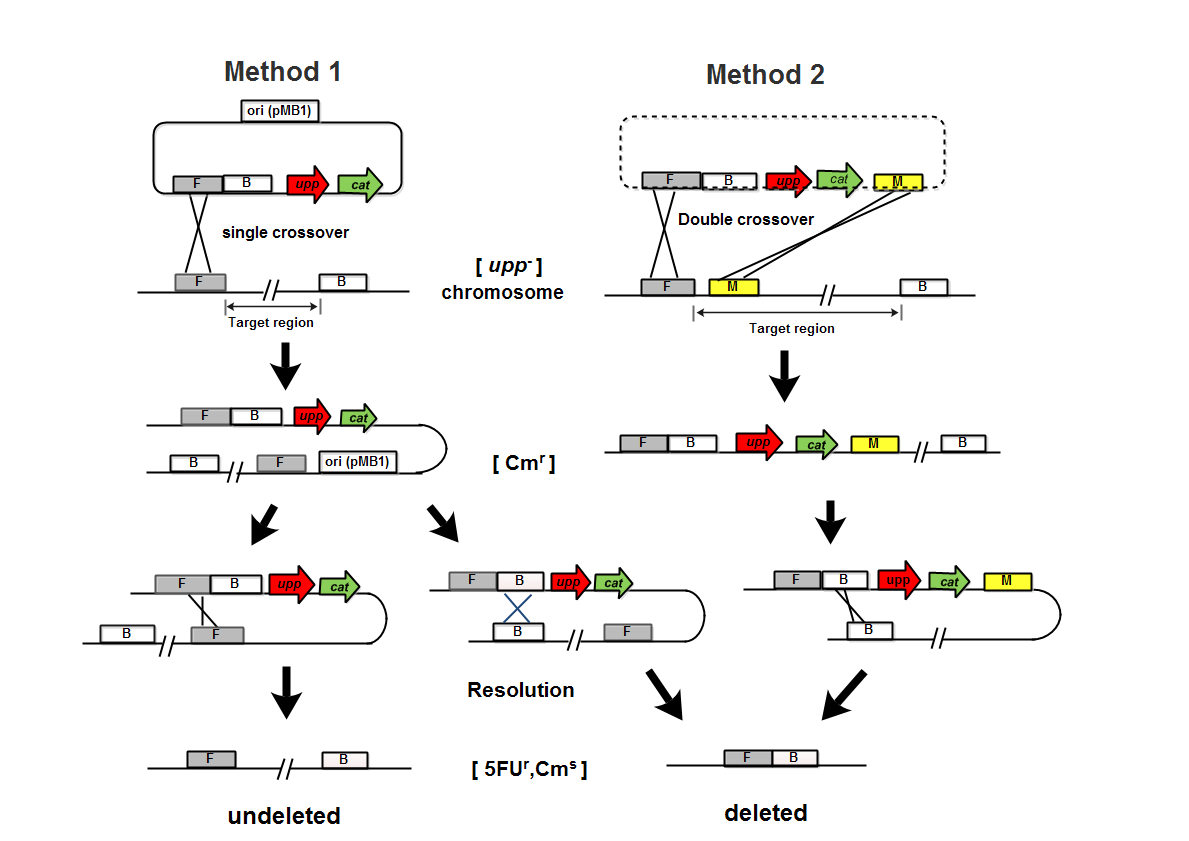
**

# Figure S4 Schematic representation of the scarless deletion methods for genome streamlining.

F and B, homologous arms flanking the target regions to be deleted; M, an internal sequence in the target region; *cat*, chloramphenicol resistance gene; *upp*, the gene encoding uracil phosphoribosyl-transferase.

Method 1: The suicide plasmid containing the upstream and downstream sequence was introduced into the recipient strains and integrated into the chromosome of recipient cells after a single crossover event. The transformants growing on chloramphenicol plates were transferred into LB liquid medium without chloramphenicol and cultivated for about 6 h. Subsequently, the cultures were spread on MM medium plates (5FU) and cultivated for 24 h. Cells in which another single crossover event occurred, corresponding two kinds of strains, with or without the target region, can grow on the MM medium plates (5FU). The two kinds of strains can be differentiated using colony PCR.

**Figure S4 (continued)**

Method 2: The linearized plasmid, which contains the upstream sequence, downstream sequence, and an internal sequence of the target region, as well as a cat-*upp* cassette, was introduced into cells of recipient strains. The DNA fragment was inserted into genome of recipient cells when a double crossover event occurred. Transformants growing on chloramphenicol plates were transferred into LB liquid medium without chloramphenicol and cultivated for 6~12 h. Subsequently, the cultures were spread on MM medium plates (5FU) and cultivated for 24 h. If a single crossover event occurred during the second cultivation step, the resulting strains without the target region can grow on MM medium plates (5FU), and the deletion can be further verified by colony PCR.

**Supplementary tables:**

# Table S1 Regions deleted in the course of genome reduction

| Deletion units | Genes | Position (start-end) | Amount of DNA removed (bp) | Gene *  Position (start-end)* | Amount of DNA removed (bp)* |
| --- | --- | --- | --- | --- | --- |
| pro1 | *glmS-ybdG* | 202079-220159 | 18080 | *ybbU-ybdE* | 17904 |
| pro2 | *ydcL-ydeJ* | 530913-569136 | 38223 | *ydcL-ydeJ* | 40491 |
| pro3 | *ydiM-ydjC* | 653427-664247 | 10820 | *ydiM-ydjC* | 11392 |
| pro4 | *yjcM-yjdJ* | 1263862-1277973 | 14111 | *yjcM-yjdJ* | 15793 |
| pro5 | *ynxB-yncE* | 1880238-1899909 | 19671 | *ynxB-dut* | 20485 |
| pro6 | *yoaV-yobO* | 2045931-2078102 | 32171 | *yoaV-yobO* | 32218 |
| spβ | *yotN-yokA* | 2152450-2286236 | 133786 | *yotN-yokA* | 134406 |
| *Skin* | *arsB-sknR* | 2656047-2700732 | 44685 | *SpoIVCB-spoIIIC* | 48765 |
| PBSX | *ykdA-xlyA* | 1320637-1348133 | 27496 | *ykdA-xlyA* | 27518 |
| *pps* | *ppsD-ppsA* | 1964049-1990227 | 26178 | *ppsE-ppsA* | 37772 |
| *pks* | *pksA-aprX* | 1782034-1863389 | 81355 | *pksA-ymaC* | 81610 |
|  | *ycxB-sipU* | 404543-454472 | 49929 | 404030-454150 | 50121 |
|  | *ydeK--ydhU* | 570428-634517 | 64089 | 569556-633930 | 64375 |
|  | *yisB-yitD* | 1148354-1173832 | 25478 | 1147460-1173400 | 25941 |
| Spβ’ | *cgeE-yodU, ybqP-ypmR* | 2145225-2152449; 2286237-2291291 | 12280 | 2145212-2291480 | 11863 |
|  | *yrkS-yraK* | 2702180-2750426 | 48246 | 2700983-2749579 | 48597 |
|  | *lytH -yurT* | 3321811-3354462 | 32651 | 3320412-3353961 | 33550 |
|  | *sboA-ywhH* | 3836658-3847468 | 10810 | 3835087-3846844 | 11758 |
|  | *pdp-rocR* | 4049079-4147089 | 98010 | 4048077-4146157 | 98081 |
|  | *yybP-yyaJ* | 4169247-4195609 | 26362 | 4168190-4194768 | 26579 |
|  | *yeeK-yesX* | / | / | 752602-773317 | 20716 |
|  | *yncM-yndN* | / | / | 1902720-1916311 | 13592 |

* non-essential regions deleted by Morimoto *et al*. (2008)

# Table S2 Primers used in this study

| Primer name | Primer sequence (5’-3’) |
| --- | --- |
| Primers for pU-pro1 | |
| glmS-for | GCGAGTGAATTCTCCTACAACATGC |
| glmS-rev | GAAAGGTGTACATTTGGAGCTACTCCACAGTAACACTCTTCGCAAG |
| ybdG-for | AAGAGTGTTACTGTGGAGTAGCTCCAAATGTACACCTTTCCGTAAG |
| ybdG-rev | CCCTTCATCCTAGCGAACCTTTCTC |
| glmS- ybdG-for | TCTGGTACCCAACATGCCGCTTCTGTCT |
| glmS- ybdG-rev | CATGTCGACGCGAACCTTTCTCATAAACTCC |
| glmS-d-for | AGTCTCGAGCGAAAGAACGTCTACAGTGG |
| glmS-d-rev | GGCAGATCTAGACCTGTTGAGCAGTGG |
| Primers for pU-pro2 | |
| ydcL-for | GCCGCATCAATGATACCAAGGG |
| ydcL-rev | CCATACCATTGAGCCATGAATTAGGGCATTACTTTCTCCATAAAAGATTG |
| ydeJ-for | GCCCTAATTCATGGCTCAATGGTATGGCTCATTACATTTAGAATG |
| ydeJ-rev | TGGCTTCGTGTTCTGGTATCGAG |
| ydcL-ydeJ-for | TGCGGTACCGAGTAAGATCGCTATCCTTGC |
| ydcL-ydeJ-rev | GTCTCTAGAACCTGCCGCCATTATCAAATC |
| ydcLd-for | GTGCTCGAGTGTTGGAGGTTCTCGTTG |
| ydcLd-rev | CGCAGATCTGCTCTACTCACCTTACCTTG |
| Primers for pU-pro3 | |
| ydim-for | CTGCTGCAGTAATGCCTACTCCTGCCT( |
| ydim-rev | TATGGATCCTCACCTCACATCGCTTATCGT |
| ydjc-for | ATGGGATCCTCATAGTCGGAGTAAGCCTTG |
| ydjc-rev | GATGGTACCTACCTGCTGGGACGGAACT |
| Primers for pU-pro4 | |
| yjcM-for | AAGCAGTTGACGAATAGAAGGT |
| yjcM-rev | GATAGCCAGTTACCGCCTATGCTGATGGTTGGATGGAT |
| yjdJ-for | CAACCATCAGCATAGGCGGTAACTGGCTATCTTC |
| yjdJ-rev | GTAAAGCTCAGGCAAACAGATG |
| yjcM-yjdJ-for | TATGGTACCGGAGCAACAGTAACGAGTAAAG |
| yjcM-yjdJ-rev | ATAGTCGACAGGCATCAGGCATAAATGGAT |
| yjcMd-for | CGTACTAGTCAAGCATCTGTATAGGGTTTCC |
| yjcMd-rev | ATTATGACGTCGTCTATCATTGTCACCATTAGTGT |
| Primers for pU-pro5 | |
| Pro5A-for | TATGGTACCGCAGTCCGCTCTTGTGATG |
| Pro5A-rev | TAGCTATTATCTGC*GGATTGAAGCAAGCATGGTTATAATTCCC* |
| Pro5c-for | GCTTGCTTCAATCC*GCAGATAATAGCTACGTTTCGATTTACTGTAAAAACA* |
| Pro5c-rev | ATCGTCGACTTATTCCCTGGCGACATACC |
| Pro5B-for | CGGCTCGAGTCTACTTGGAACTGGTCTTGT |
| Pro5B-rev | TAGACGACGTCCATGACATATCCTCCATGATG |
| Primers for pU-pro6 | |
| yoaV -for | CGTGGTACCGGCAGAGATGGATGAATCTT |
| yoaV -rev | CTAAAGAAGAACTGAATAATGAAGCCGCCTTTCTTGTTAAAATATCG |
| yobO -for | AGGCGGCTTCATTATTCAGTTCTTCTTTAGGTGGATATGGAGAGTAT |
| yobO -rev | TGATCTAGAGTCTTTGCGTATTGAGGCGATGC |
| yoaV - yobO -for | GATGGTACCACAAGCTCCCAAATAGAGTG |
| yoaV - yobO –rev | AGTTCTAGATTGCGTATTGAGGCGATGC |
| yobA-for | GACCTCGAGGAATCCTGACGACAGCCTATT |
| yobA -rev | TGCGTGACGTCCTCTTGACGAATGTACTCTT |
| Primer for pU-skin | |
| Ska-for | CCCTTGGGTAATGCTATGTAACAGCT |
| Ska-rev | CTAAGGAGATGGAAGTTGTCCGAGTTGCGATTCCA |
| Skc-for | CAACTCGGACAACTTCCATCTCCTTAGACGCATTTTCCTATGA |
| Skc-rev | TTCGCCTTCTTCCGCTTTTCCT |
| Skac-for | TATGGTACCGCTGTTTGTCCAAATGAGGAG |
| Skac-rev | TATGTCGACTGCGTGAGATACCGAGTTCTT |
| Skb-for | ATACTCGAGCATACCATTCTTTGCCCTTCAC |
| Skb-rev | CGCAGATCTACTTTAAATGTCGCAGATGAGG |
| Primers for pU- spβ |  |
| yotN -for | TGGGGGTACCATATACAGGTGATTACCGTGAG |
| yotN -rev | TGAGCTTGATTCAATGAAGTAAACAAAGCCAGTATCCCGATTG |
| yokA -for | TGGCTTTGTTTACTTCATTGAATCAAGCTCAGAGAAAAAGAACGAG |
| yokA -rev | GCCAACTGCAGAGAATCCAGCACATTATCAG |
| yotN-yokA -for | CAGGGGTACCCAGGTGATTACCGTGAGG |
| yotN-yokA -rev | GGCGCTCTAGAAATCCAGCACATTATCAG |
| yotM-for | ACTACTCGAGCGGCTCTCTAAATTGAAACAG |
| yotM-rev | TGCAGACGTCGTGGAGGTATTATGTGTAATC |
| Primer for pU- spβ’ |  |
| spβ' A-for | AGCGTTCCCTGAGGTGAAAAT |
| spβ' A-rev | GGCATAAAGGACGCAGATTCATTGAGCGGTATCCAGCTATC |
| spβ' C-for | GGATACCGCTCAATGAATCTGCGTCCTTTATGCCTTTTTGAAC |
| spβ' C-rev | GCCCTCCTTTGTAATCGTTAATGG |
| spβ' AC-for | CTAGGTACCTCCGGCTACTGGATTGGAAC |
| spβ' AC-rev | AGCTCTAGATGCTCAGTATCGAATACAAACA |
| spβ' B-for | ATTCTCGAGGAACCCTTCGCATAACTGACAACC |
| spβ' B -rev | ACTAGATCTGTCTGAATCGGCATCGTTTGAT |
| Primers for pU- PBSX | |
| PBSX-A-for | CGATATGTGGAAGCCGTCAAG |
| PBSX-A-rev | TTTCGTCGCAGGCCCGTAGATAGGCGATCAGCAGCACTT |
| PBSX-C-for | CTGCAAGTGCTGCTGATCGCCTATCTACGGGCCTGCGACGA |
| PBSX-C-rev | ATGGTCTGGTGCATCGTGG |
| PBSX-AC-for | TACGGTACCAGGGCGGATGCTAAGTATAAAG |
| PBSX-AC-rev | CGCTCTAGACTGGGAACGGTATCTCATTGTC |
| PBSX –B-for | GTACTCGAGAGCCGCTTCTGAAATTGAATAC |
| PBSX –B-rev | CAGAGATCTCGGTAAACGAAATGGTGCC |
| Primers for pU- PKS | |
| PKS-A-for | GTGAGTGGGTCTATGATTAAACCTACAGCA |
| PKS-A-rev | GTTTAGCCAATAGGTAATGACAATCCTGCTTCCTTTGCAATAT |
| PKS-C- for | AGCAGGATTGTCATTACCTATTGGCTAAACCTCATATAATGAACCG |
| PKS-C- rev | TCGTCTTGTGAAGCATCATGTTTAAGCA |
| pKS-AC- for | TGAGGTACCGAAGCGTATGTGATGCCAAGT |
| pKS-AC- rev | GGCTCTAGAGACAGAGGGACGGTGAATGA |
| PKS-B-for | GACCTCGAGACACAGGATGAATTGCTTGCTT |
| PKS-B-rev | TATATGACGTCGCCAAATGAGTGACCAGGATAC |
| Primers for pU- PPS | |
| ppsE-for | CACTGTCGACTCTGCTTGTTCGCGAAAT |
| ppsE-rev | CAATCGGCACAAATGGATCCCTAATGAATCCGTGAAGAAAGGT |
| ppsA-for | TCATTAGGGATCCATTTGTGCCGATTGTGCCATCAA |
| ppsA-rev | TAATGGTACCGGCATCTCAAGCGGACCAGG |
| ppsEA-for | TAGAGGTACCTTGCTCGGATACTTGAACTTGA |
| ppsEA-rev | TATAGTCGACATCATTACAACCAGTCCGTCAT |
| ppsE2-for | TTATCTCGAGGCGTCCTCGGTCAGTTCTT |
| ppsE2-rev | TATTGACGTCTTGTACGGCCTTCAGATGATTG |
| Primers for pU- ycxB-sipU | |
| ycxB-u-for | ACCGTTTCAGTGTCAGAAAAATTATGGC |
| ycxB u-rev | TGAGACGGTTATCCGAAGAGGAGATGAACAGATTCAAGGAG |
| sipU-d-for | CATCTCCTCTTCGGATAACCGTCTCAATTCACTGGATAGCAG |
| sipU-d-rev | TCTCCAACTGATGCCTTGGCAT |
| ycxB-sipU-for | CTGGGTACCTCCTTATTCACCGAATGTTC |
| ycxB- sipU-rev | TGAGTCGACTCTCCAACTGATGCCTTG |
| ycxB-d-for | GTCCTCGAGGCCACTTGATGTATTGACC |
| ycxB-d-rev | CAGTAGACGTCACTCGTCCTCCTCTGAAG |
| Primers for pU- yisB-yitD | |
| yisB-for | ATATGAAGAACTGCAAAGCGCCCA |
| yisB-rev | TGGCTTCTGTAATCTGAGTTCAGCCATTCCATTTAAACGTACAG |
| yitD -for | TTAAATGGAATGGCTGAACTCAGATTACAGAAGCCAGAGAAAGCGG |
| yitD -rev | AAGGAATGGTCCACATCCCGAAAG |
| yisB-yitD-for | TATGGTACCCAAGAGGCGAAGGGCAGAT |
| yisB-yitD-rev | TCAGTCGACAGGCATTGGCACAGAGATC |
| yisB-d-for | GACCTCGAGAAACAGCCGCCTGAGAAG |
| yisB-d-rev | CAGTAGACGTCTGACAGCAGACTTGACGATAC |
| Primers for pU- pdp-rocR | |
| pdp-for | CTGGGTACCCCTGACAGCAGTGTATGGA |
| pdp-rev | AATTTCTTCAGCCGATATTGGCTATGACAACATCCGCATC |
| rocR-for | GATGTTGTCATAGCCAATATCGGCTGAAGAAATTTTCTCAC |
| rocR-rev | TGAGTCGACAGTCGCTGATTTCGGAAATTC |
| pdp-rocR-for | CCGGGTACCTGTATGGATGTGGAGTGC |
| pdp-rocR-rev | TCTGTCGACCTTGAGTTTGCAGGCTCTG |
| pdpu-for | GTCCTCGAGAAGCGTTACGAGCGGTTC |
| pdpu-rev | CAGTAGACGTCGGCTATACAGATGGAAGCATT |
| Primers for pU- yybP-yyaJ | |
| yybP-for | GCAGCAAGACGTGTTAAACTATAT |
| yybP-rev | CCGACAAGCAATGAGCCGAATTGGAGCAGTCAGACCT |
| yyaJ-for | ACTGCTCCAATTCGGCTCATTGCTTGTCGGATTTATT |
| yyaJ-rev | GGCGAAGGAGGAGGAAGAAATGC |
| yybP-yyaJ -for | TATGGTACCACCCAGATACAGATACGATTTG |
| yybP-yyaJ-rev | CGGTCTAGAGGCGAAGCGAATGATTTGT |
| yybP-d-for | CTCCTCGAGTTAAGCGATACCTTGCCTTCC |
| yybP-d-rev | ACGAGATCTGCTCCATATTGTCATTCCAACC |
| Primers for pU- yrkS-yraK | |
| yrkS-for | GCTTCGCACTTAAACACAGCGG |
| yrkS-rev | CTAACATGTCGTGTAACCTCTCAGTATTACGCAGCATTACCCAAGGGGTGATG |
| yraK-for | CCCTTGGGTAATGCTGCGTAATACTGAGAGGTTACACGACATGTTAG |
| yraK-rev | TGCGGCTCAGTATTATACGGTTTCAAC |
| yrkS-yraK-for | CAGGGTACCAGCCGAACTGAACAATGG |
| yrkS-yraK-rev | TATGTCGACGCGGTGCTCCTGTCAACA |
| yrkS-d-for | GTGCTCGAGCTGTTTGTCCTAATGAGGTG |
| yrkS-d-rev | GACAGATCTGAGGAGCTTGGTCGTGTATT |
| Primers for pU- ydeK-ydhU | |
| ydeK-for | AGCAAGTTACTACACTCCAAAC |
| ydeK-rev | TTAGATGAATCAGGCGGCAGTGAATATCGCCGTTATCATGTGC |
| ydhU-for | GCGATATTCACTGCCGCCTGATTCATCTAATAAAGCATTGAT |
| ydhU-rev | CATCGTTGCCTTGGTGAGCC |
| ydeK-hU-F | GTAGAGCTCGCTGGCACTTGTATCATCATTA |
| ydeK-hU-R | CGCTCTAGAGCTTGGAAGGCTGAGGTT |
| ydeK-d-for | GATCTCGAGTCTCGTGCAGAATCACTGAGG |
| ydeK-d-rev | CTATCGACGTCTTGAATGGGTGTTGTGCTTGG |
| Primers for pU- lytH- yurT | |
| lytH-for | AATTGTTGTGAACGGGCTTT |
| lytH-rev | CTCTTCTTCTGTCTCTTGATTGTTGCTGACCTCGGCTTTC |
| yurT-for | GAAAGCCGAGGTCAGCAACAATCAAGAGACAGAAGAAGAG |
| yurT-rev | CTATATCACGCAACCATACAGT |
| lytH- yurT-for | CGAGGTACCAATTGTTGTGAACGGGCTT |
| lytH -yurT-rev | GAGGTCGACGACTCGGCGGCTTATATTAG |
| lytHd-for | GCGCTCGAGTCAGCCAATACTTACTGTC |
| lytHd-rev | CATCAGACGTCTGAAGGTCATCATTCCATTT |
| Primers for pU- sboA-ywhH | |
| SboA-for | TGTTATCGGTGTCTCTTGCTTCAT |
| sboA-rev | AGGACAGGGACTTGGCGATTCTAGGCTTCCGCTCGTGGTGATTGTA |
| ywhH-for | TACAATCACCACGAGCGGAAGCCTAGAATCGCCAAGTCCCTGTCCT |
| ywhH-rev | GGGCCGTCCGGTTTATGTCA |
| sboA-ywhH-for | TATGGTACCGCCGGTGCAACAGGTCTATTC |
| sboA-ywhH-rev | TATGTCGACGGGCCGTCCGGTTTATGTC |
| sboAd-for | CCGAGATCTACTGACGCACCGCTGTAAT |
| sboAd-rev | TACTAGACGTCTCCCTGCCTCTTCCGAAATC |
| Sy-t-for | GGGCGGCGTGTTAGAAATC |
| Sy-t-rev | CGAAGACAGACATAGCGACTC |
| Primers for pCU- purA | |
| purA1-for | TATGTCGACGTGAAAGGGCGGTGTCTT |
| purA1-rev | GCGTCTAGAGCGAATATGGAACAACAACAG |
| purA2-for | CGGTCTAGACTCTTCTTCCACTTCATCCAAT |
| purA2-rev | TGAGGTACCCATCCCGACAGCCTTCTT |
| Primers for pHP13- ppg | |
| prs-for | AAAGGTACCAGAGAACAAGGAGGGGGTTTATCCATGTCTAATCA |
| prs-for | GACGTCGACAGATAATCTAGATTGTCGTTTTAGTTGTCCTATTAC |
| purF-for | ATATCTAGAATATTAAGAGGAGGAGGCTATCCCATGCTTGCTGAAATCA |
| purF-rev | TTTGTCGACGCCGAACTGCGCTGCCTTTATGTC |
| guaB-for | TATGTCGACATATTAAGAGGAGGAGGGGGATTTACTAATGTGGG |
| guaB-rev | CCTGGATCCGTACGACCTCCGTATTTCAT |
| Primers for pCU- tdk | |
| tdkA-for | GCAGTCGACTTCAGTCAGCAAGCCTCTGTAATCAA |
| tdkA-rev | CGAGGATCCCATCATCACGAAGTCCCAGGTAAATC |
| tdkC-for | TATGGATCCCCACCCGCTTTGTTTCATTATGTA |
| tdkC-rev | TCAGGTACCCGGCTCTTACGCCACTTTATCC |
| Primers for pHP13- untd | |
| ushA-for | TAGAGCTGCAGCCAAGGAGGGTATAGCTATGAAATTATTGCAGCGGGG |
| ushA-rev | TACAGTCGACTTACTGCCAGCTCACCTCACC |
| ndk-for | TACAGTCGACCCAAGGAGGGTATAGCTATGGCTATTGAACGTACTTTTT |
| ndk-rev | CAGGGATCCTGGCAGCTCTAGATTAACGGGTGCGCGGGCACA |
| thyA-for | GCAGCTCTAGACCAAGGAGGGTATAGCTATGAAACAGTATTTAGAACTGATGC |
| thyA-rev | CAGGGATCCTTAGATAGCCACCGGCGCTT |
| dut-for | CAGGGATCCCCAAGGAGGGTATAGCTATGAAAAAAATCGACGTTAAGATTCT |
| dut-rev | TCCCCCGGGTTACTGACGACCAGAGTGACCAAA |
| Primers for RT-PCR | |
| RTPrs-for | CGATTACACTTGCTGCTAATGC |
| RTPrs-rev | GCTGACTGATTGCTGCTCAT |
| RTpurF-for | GCAGAGGCAACAGGCATTC |
| RTpurF-rev | ACGATAGAGTCATCCACCATCA |
| RTguaB-for | GCTCACGGACACTCTCAAG |
| RTguaB-rev | GCTTCAGTCGCACAATCATAA |
| RTushA-for | TGACTTACAGGATGCCGAAC |
| RTushA-rev | CACTTTTCCTGCTGGCGT |
| RTndk-for | TGGCTTTTATGCTGAACACGA |
| RTndk-rev | CCAGCACGGAAACCACGAT |
| RTthyA-for | TTCCATCATCCACGAACTG |
| RTthyA-rev | TTTTCATCGGCCCATTCGTC |
| RTdut-for | ATTGGGACATAAGCACGGTA |
| RTdut-rev | CCAAACGGAAATCATCAACTGG |
| RTrrnA_for | ATGCGTAGAGATGTGGAGGAA |
| RTrrnA_rev | GCGGAGTGCTTAATGCGTTA |
